# A graphical model approach visualizes regulatory relationships between genome-wide transcription factor binding profiles

**DOI:** 10.1093/bib/bbw102

**Published:** 2016-10-25

**Authors:** Felicia S L Ng, David Ruau, Lorenz Wernisch, Berthold Göttgens

**Affiliations:** Department of Haematology, Wellcome Trust and MRC Cambridge Stem Cell Institute & Cambridge Institute for Medical Research, Hills Road, Cambridge, UK

**Keywords:** ChIP-seq, network, transcriptional regulation, haematopoiesis

## Abstract

Integrated analysis of multiple genome-wide transcription factor (TF)-binding profiles will be vital to advance our understanding of the global impact of TF binding. However, existing methods for measuring similarity in large numbers of chromatin immunoprecipitation assays with sequencing (ChIP-seq), such as correlation, mutual information or enrichment analysis, are limited in their ability to display functionally relevant TF relationships. In this study, we propose the use of graphical models to determine conditional independence between TFs and showed that network visualization provides a promising alternative to distinguish ‘direct’ versus ‘indirect’ TF interactions. We applied four algorithms to measure ‘direct’ dependence to a compendium of 367 mouse haematopoietic TF ChIP-seq samples and obtained a consensus network known as a ‘TF association network’ where edges in the network corresponded to likely causal pairwise relationships between TFs. The ‘TF association network’ illustrates the role of TFs in developmental pathways, is reminiscent of combinatorial TF regulation, corresponds to known protein–protein interactions and indicates substantial TF-binding reorganization in leukemic cell types. With the rapid increase in TF ChIP-Seq data sets, the approach presented here will be a powerful tool to study transcriptional programmes across a wide range of biological systems.

## Introduction

Transcription factors (TFs) are an important class of proteins that bind to cis-regulatory elements and control the transcription of nearby genes. They have long been recognized as important regulators of haematopoietic cell-type identity and, therefore, have been extensively studied to gain a better understanding of their role in the differentiation of normal blood stem cells [1–7]. With the advancement of chromatin immunoprecipitation assays with sequencing (ChIP-seq) technology in recent years, both large-scale consortium efforts and individual laboratories have contributed to an immense collection of public ChIP-seq data sets that include samples from all stages of haematopoietic development [8–11]. However, the precise mechanisms by which TFs influence cell-type identity are still largely unknown, and analysis across large numbers of ChIP-seq experiments is made difficult by an even larger number of genome-wide binding sites. The binding of distinct TFs in the same cell type is also known to be highly correlated and, therefore, correlation or enrichment analysis alone is insufficient to identify relevant TF interactions. Few studies have addressed the impact of global TF binding during blood cell development in part because of a lack of suitable methods for integrating large-scale data sets. The development of alternative approaches for the comparison of genome-wide binding sites therefore has the potential to provide useful biological insights.

In this study, we used a graphical modelling approach to create a network that displays the relationship between ChIP-seq samples and TF combinations, whose correlated binding on regulatory elements is most likely causally linked. Graphical models were used to infer the joint probability distribution of a set of samples to distinguish ‘direct’ from ‘indirect’ dependence between TFs and, therefore, serve as a useful approach to understanding combinatorial regulatory interactions. In the past, graphical models such as Bayesian networks and Gaussian Graphical Models (GGMs) have been used to infer networks from gene expression data such as microarray. However, these studies have mainly focused on data from lower eukaryotes where data sets are smaller and regulatory interactions are less complex [12–14]. Here, we show that a similar approach can be applied to genome-wide TF-binding profiles of a large-scale haematopoietic ChIP-seq data set and demonstrate the usefulness of a network-based visualization of ChIP-seq samples over existing methods such as correlation heatmaps, mutual information or enrichment analysis. In general, the network provides valuable insights into TF interactions that are supported by known protein–protein interactions in normal haematopoietic development as well as the reorganization of TF binding in leukemic cells.

## Materials and methods

### HAEMCODE ChIP-seq data source and processing

Public mouse ChIP-seq data sets from blood-related cell types were obtained from the NCBI Gene Expression Omnibus and the EMBL-EBI European Nucleotide Archive (Supplementary Table S1). Raw data were processed and peaks were discovered using a standardized pipeline as described in Sanchez-Castillo *et al.* [11]. Collectively, there were 501 163 genomic segments from significant peaks (overlapping regions  ≥1 bp merged) identified in 367 ChIP-seq samples. We use the term ‘sample’ to refer to one ChIP-seq profile. If multiple different TFs were immunoprecipitated in a single study, the individual TFs are therefore counted as individual samples.

### Multi-sampleTF-binding profile

Given a set of ChIP-seq samples P=[1,p] with N=[1,n] genomic segments (peaks), a binary matrix of TF-binding profiles, X, was constructed using the intersectBed function in BEDTools [15] and consists of n rows of genomic segments and p columns of ChIP-seq samples. Each element in the matrix, $X_{ij}\;(1\leq i\leq n,\;1\leq j\leq p)$, takes the value of ‘1’ if a peak was identified in the corresponding sample and ‘0’ otherwise. In this study, n=501,163 and p=367.

### Heatmap and hierarchical clustering of ChIP-seq samples

Using the multi-sample TF-binding profile described above, we calculated the Pearson correlation coefficient for all pairwise comparison of samples. Hierarchical clustering was then applied to the matrix of correlation values using the *hclust* function in R [16], and a clustered heatmap was generated using the *gplots* package [17] in R.

### Network inference algorithms for constructing a TF association network

The multi-sample TF-binding profile described above was used to generate a ‘TF association network’ G=(V, E), where V is the set of vertices (i.e. nodes) and E is the set of undirected vertex pairs (i.e. edges). Each node corresponds to a single ChIP-seq sample or TF, and an edge in the graph denotes a significant ‘direct’ dependence between the binding of a pair of TFs. Direct dependence was determined using four algorithms—Bayesian network, Gaussian graphical model, graphical lasso and linear regression—using the *bnlearn* [18], *GeneNet* [19] and *glasso* [20] R packages as well as the *TIGRESS* [13] MATLAB software. See Supplementary Methods for more details.

To obtain a consensus network for further analysis, we constructed a new network based on the edges that were discovered in any three or more algorithms, and the resulting graph consists of 362 nodes and 1182 edges. Cytoscape version 3.2.1 was used to visualize the network [21]. Source code for generating the consensus network in this study can be downloaded at (https://bitbucket.org/feliciang/publication_tf_association_network).

### Protein–protein interaction data and performance testing

Known and predicted protein–protein interactions were downloaded from the STRING website [22] (http://string-db.org/). Performance of the ‘TF association network’ was compared against the correlation graph and each of the individual network inference algorithms by comparing the precision and recall rate of identifying mouse protein–protein interactions in the database. In total, there were 56 unique TFs in our data set that have evidence for protein–protein interactions in STRING. Edges of the network were annotated with high-confidence protein actions (keyword ‘binding’) to highlight physical interactions (thick black lines on network) rather than functional interactions (thick grey lines). Interactions were considered high-confidence links if the combined score was  ≥500.

## Results

### Comparison of multiple genome-wide ChIP-seq binding profiles demonstrates that improvement of the current analysis methods is needed

**Figure 1.**
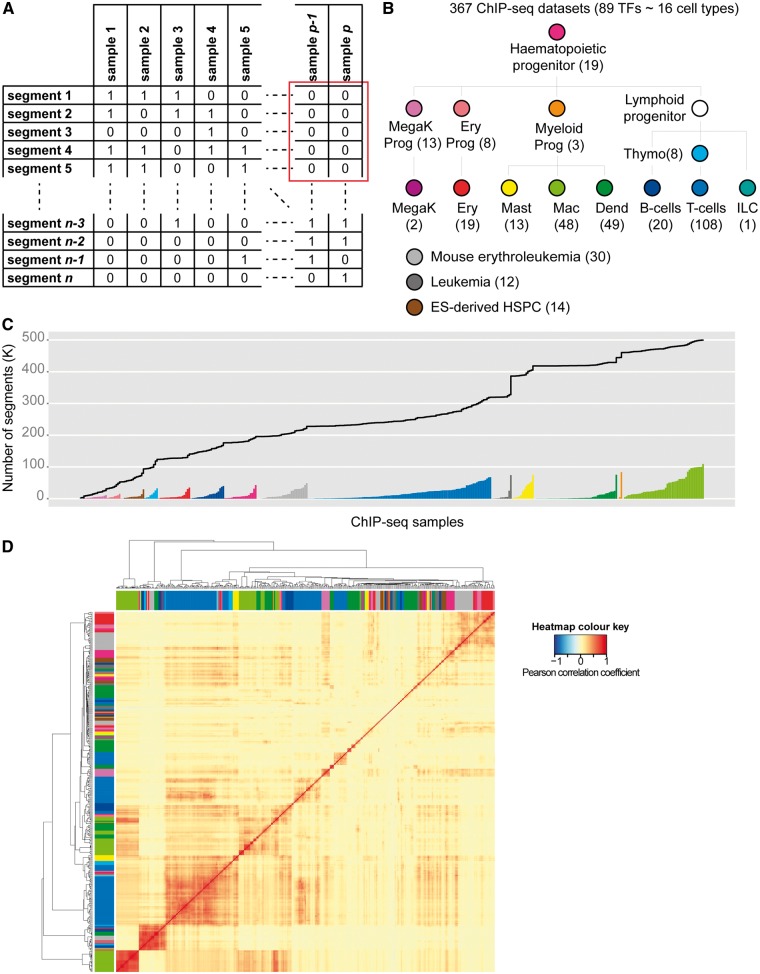
Multi-sample comparison of genome-wide TF-binding profiles. (**A**) Multi-sample TF-binding profile matrix. TF ChIP-seq peaks from multiple samples can be represented in a binary matrix where rows are ***n*** collective genomic segments from peaks identified in ***p*** samples. Each element in the matrix can be either a ‘1’ denoting a peak or ‘0’ denoting absence of a peak in that particular sample. (**B**) ChIP-seq samples used in this study (total=367) encompass all major blood cell types as well as other haematopoietic-related cell types/lines. Numbers of samples for each cell type are shown in brackets. (MegaK Prog—megakaryocyte progenitor, Ery Prog—erythroid progenitor, Myeloid Prog—myeloid progenitor, Thymo—thymocytes, MegaK—megakaryocyte, Ery—erythroid, Mac—macrophage, Dend—dendritic cells, ILC—innate lymphoid cells, ES-derived HSPC—embryonic stem cell-derived haematopoietic stem/progenitor cells) (**C**) Barchart indicates the number of peaks for each sample grouped and coloured by cell type as in B. Line plot shows the cumulative number of unique peaks. (**D**) Heatmap and hierarchical clustering of 367 ChIP-seq samples. Each element in the heatmap indicates the pairwise Pearson correlation coefficient between samples in the corresponding row and column. Colour bars indicate the cell type as in B that each sample belongs to.

Recent research on genome-wide TF-binding profiles increasingly relies on larger sample sizes (some in the order of tens or even hundreds of ChIP-seq samples) to uncover novel biological insights. Bioinformatics analysis therefore often requires analysis of a data matrix that may include hundreds of thousands of genomic regions and hundreds of ChIP-seq samples containing information about where each TF binds in the genome. This information is commonly stored as a binary matrix where ‘1’ denotes the presence of TF binding and ‘0’ otherwise (Figure 1A). Each row in the matrix corresponds to a genomic segment bound by at least one sample in the compendium, and each column corresponds to a sample. The term sample is used here to refer to one ChIP-seq profile. Hence, the set of *n* genomic segments is the union of all TF-bound sites in *p* samples. Using this ‘multi-sample TF-binding profile’, one commonly used technique to understand the relationship between ChIP-seq samples is to calculate the correlation coefficient between all pairs of samples, which are then displayed in a clustered heatmap. This provides a measure of similarity between the global binding profiles of one TF against all the other TFs in the study. However, as the numbers of samples increase, the number of nonoverlapping segments increases as well, especially with increasing diversity in sample conditions. This can have unintended consequences on the calculation of correlation coefficient because the vectors of zeroes (see red box in Figure 1A) contribute to increased correlation, even though these binding sites are often irrelevant to a comparison between two samples. Discarding these entries, however, will give an inaccurate estimate of the correlation between a pair of samples in comparison with all the other samples.

As the available data increase, improved methods are needed to compare and discover relationships between ChIP-seq studies, and here, we present a new approach to this problem. In particular, the joint probability distribution of the entire data set is more informative than pairwise comparisons using correlation coefficient. We applied our new technique to public data sets in a previously reported ChIP-seq resource—the HAEMCODE repository, now part of the CODEX compendium [10, 11]—and at the start of this study, this consists of 367 uniformly processed ChIP-seq samples (a subset of which is data published by our laboratory). These data sets encompass 86 different TFs covering all major haematopoietic or haematopoietic-related cell types (Figure 1B). First, we constructed a ‘multi-sample TF binding profile’ matrix (see the ‘Materials and Methods’ section) to store information about TF-binding locations in the genome for all 367 samples. Matrix elements take the value of ‘1’ if a ChIP-seq peak was identified by our data processing pipeline and ‘0’ otherwise. As shown in Figure 1C, each sample contributes to increasing numbers of genomic segments (additional rows in matrix), which sums up to 501 163 unique TF-binding segments for 367 samples. Importantly, this also increases the number of genomic segments that are not binding peaks in the majority of all other samples and therefore increases the number of rows with zeroes. Using these data, we found that the majority of samples, even those from distantly related cell types, are (to some degree) positively correlated with each other (Figure 1D), as the minimum correlation coefficient observed between a pair of samples was 0.129, and no negative correlation was observed in any pairwise comparison. This limits the utility of correlation heatmaps to provide valuable insight into the cooperative nature of TF binding, as a threshold cannot be clearly defined from the range of positive correlation values to distinguish ‘direct’ from ‘indirect’ interactions. Moreover, correlation heatmaps are limited in their ability to show relationships between multiple samples and are not designed to distinguish likely direct from indirect links.

### Graphical models are useful for comparing ChIP-seq samples and understanding the mechanisms of TF binding

We propose an alternative method to compare and discover relationships between ChIP-seq samples using graphical modelling theory. In particular, we suggest that greater insights into TF-binding mechanisms can be uncovered using graphical models because graphs can express likely causal relationships between multiple ChIP-seq data sets. We call this type of graph a ‘TF association network’, where components of a network are nodes representing samples (i.e. TFs), and undirected edges represent causal pairwise relationships between any two TFs. The motivation for this approach stems from its widespread adoption in constructing gene regulatory networks (GRNs) from gene expression data sets such as microarray, where several concepts underlying GRN inference can be borrowed to construct a network for ChIP-seq data sets. First, the notion of conditional independence is useful for uncovering ‘direct’ interactions between TFs. Second, TF-binding networks are assumed to be sparse (few TFs interact with each other) and have an organized structure. Third, the discovery of important edges for each TF in the network can be treated as a feature selection problem by which only important TFs regulating its binding are identified. By adapting GRN algorithms, therefore, we should obtain a connected structure of TF-binding influences from a finite set of TF-bound regions in the genome.

**Figure 2:**
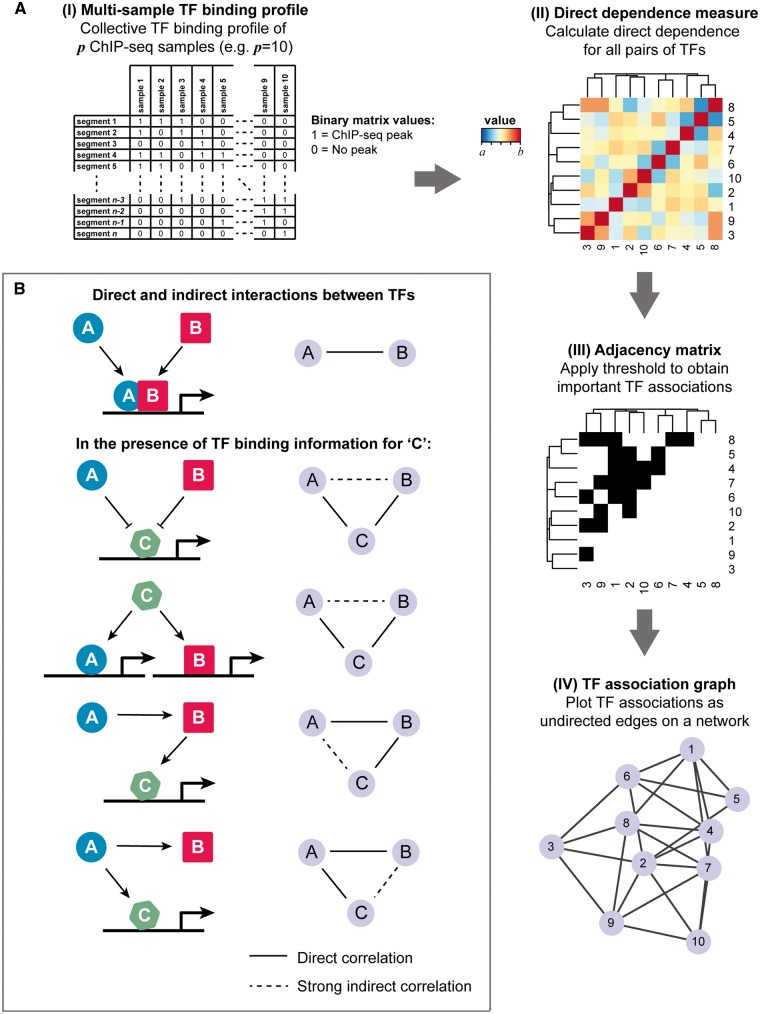
Schematic of the generation of a TF association network. (**A**) A ‘multi-sample TF-binding profile’ was used to compute conditional dependence between all pairs of samples (i.e. TFs). This produces a *p* × *p* matrix, where each element indicates the conditional dependence between the two corresponding TFs. A stronger value denotes stronger dependence between the binding profiles of the two ChIP-seq samples. Applying a threshold to the conditional dependence measure yields an adjacency matrix of the most significant ‘direct’ TF interactions, which can be displayed as edges in a network diagram. Directionality of the edges was ignored. (**B**) Example of a network involving three TFs and the difference between ‘direct’ from ‘indirect’ TF interactions. Observing strong correlation coefficients between TF *A* and *B* (top graph) does not necessarily imply ‘direct’ interactions as illustrated by the alternative scenarios (bottom four graphs).

To construct a ‘TF association network’, our aim is to identify important edges that represent likely causal pairwise relationships between any two TFs. Starting with a ‘multi-sample TF-binding profile’ (Figure 2A—I), we adapted several GRN inference algorithms to obtain a matrix of pairwise direct dependence measure for all samples in the input data (Figure 2A—II). Each element in the matrix, therefore, indicates the degree of ‘direct’ influence of the binding of one TF on the binding of another TF. In contrast, observing a large degree of correlation is not sufficient to indicate that two TFs have a ‘direct’ interaction and instead only provide weak evidence for ‘direct’ dependence. After all, in a regulatory network for haematopoiesis, we expect that most TFs are somewhat correlated with each other. To illustrate the notion of conditional independence, Figure 2B shows an example of the importance of considering ‘direct’ dependence when evaluating TF associations. In the absence of TF *C*, observing a strong correlation between TF *A* and *B* does not necessarily imply a ‘direct’ interaction between the two TFs, but may instead suggest any of the following two scenarios: (i) TF *A* and *B* independently compete with TF *C* for DNA binding, or (ii) TF *A* and *B* are commonly regulated by TF *C*. In other words, TF *A* and *B* are conditionally independent given *C* and, therefore, suggest an ‘indirect’ interaction between TF *A* and *B*, as their correlation is already explained by another TF. Furthermore, TF *A* and *B* may still be conditionally dependent given *C* in the following two scenarios: (i) TF *A* regulates TF *B*, which in turn regulates TF *C*; or (ii) TF *A* regulates both TF *B* and *C*. It is therefore crucial for network inference algorithms to be able to distinguish ‘direct’ interactions from ‘indirect’ interactions by simultaneously comparing one TF-binding profile against all other TFs. In a data set with more than three ChIP-seq profiles, conditional dependence between TF *A* and *B* indicates a likely ‘direct’ interaction between the two TFs given all the samples in the data set. This can be useful when comparing samples from multiple cell types because the joint probability distribution provides a direct comparison across all samples.

We chose one algorithm from each of the following categories to obtain the conditional dependence measures: GGM, graphical lasso, linear regression and Bayesian network. These algorithms were selected based on their performance in the DREAM5 challenge [23], reasonable computational time for our data set and for which implementation of the algorithms is readily available. Moreover, they were selected because validation and testing on gold standard data sets had been carried out to demonstrate the robustness in gene network reconstruction [23–26]. The following packages (R or MATLAB) were used to calculate the direct dependence matrix from the ‘multi-sample TF-binding profile’: *GeneNet* [19], *glasso* [20], *TIGRESS* [13] and *bnlearn* [18]. A significance threshold is then applied to the matrix, and entries that pass this threshold are given a score of ‘1’ in the adjacency matrix and ‘0’ otherwise (Figure 2A—III), with the following rules: (i) the influence of TF *A* on TF *B* is equivalent to the influence of TF *B* on TF *A*; (ii) if the direct dependence matrix is non-symmetrical, the direct dependence measure for a pair of TFs *A* and *B* is taken as the average between the influence of variable *A* on *B* and variable *B* on *A*; (iii) self-edges that denote influence of a TF on itself are ignored. Therefore, each non-zero off-diagonal element in the upper (or lower) triangle of the adjacency matrix denotes an undirected and unweighted edge between the corresponding TFs. Self-regulations were not considered because we do not assume that the binding of a particular TF induces more of the same TFs to bind. Finally, the significant edges are displayed in a graph to perform further analysis on the conditional dependence structure of TF binding (Figure 2A—IV). 

### TF association networks uncover the conditional dependence structure of TF binding and provide insights into haematopoietic transcriptional regulation

**Figure 3:**
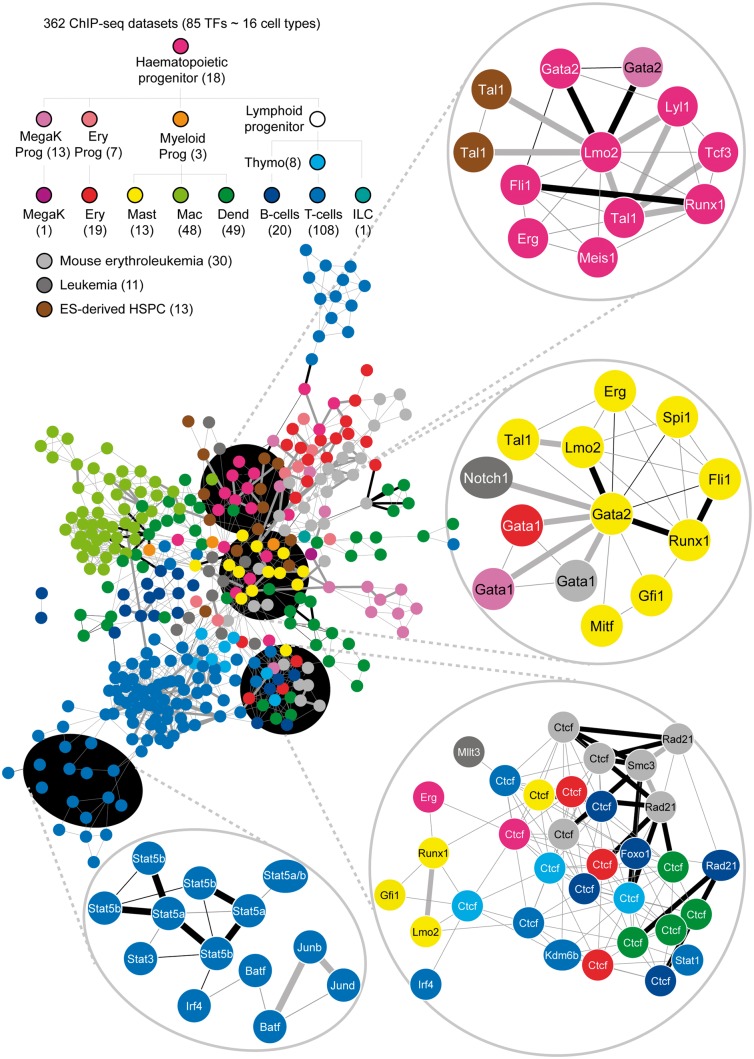
TF association network generated from 367 ChIP-seq samples. In total, there are 362 nodes (corresponding to 88 TFs and 16 cell types) in the network, each representing a single ChIP-seq sample and are coloured by the cell type it belongs to (five samples were not connected with edges to any samples in this network). The haematopoietic development tree shows the number of samples for each cell type that are included in this network. There are 1182 edges connecting the nodes, each representing a consensus edge that were discovered in at least three algorithms of four. Thick edges (black and grey) indicate high-confidence protein–protein interaction (confidence score  ≥500). Evidence for physical interactions is indicated by thick black lines. (MegaK Prog—megakaryocyte progenitor, Ery Prog—erythroid progenitor, Myeloid Prog—myeloid progenitor, Thymo—thymocytes, MegaK—megakaryocyte, Ery—erythroid, Mac—macrophage, Dend—dendritic cells, ILC—innate lymphoid cells, ES-derived HSPC—embryonic stem cell-derived haematopoietic stem/progenitor cells)

As all of the algorithms mentioned above are known to be affected by distinct strengths and weaknesses, we decided to generate a more robust network from the consensus across the four algorithms by considering only edges discovered in any three or more algorithms (Supplementary Figure S1). This resulted in a network containing 362 nodes and 1182 edges (Figure 3). Each node in the network represents a single ChIP-seq sample (i.e. TF) and is coloured by the cell type it belongs to. An edge between two nodes denotes a significant conditional dependence between the samples suggesting that interaction is likely between the two TFs. By considering only edges discovered by three or more algorithms, we obtained a list of high-confidence TF influences, as these edges are more likely to be associated with a stronger direct dependence measure (Supplementary Figure S2). Furthermore, this generates a network that connects all nodes in the graph, thus facilitating the comparison across all cell types. The graphical lasso method appeared to be an outlier because it identified many edges not present in the remaining three algorithms (Supplementary Figure S1). In comparison to GRNs, ‘TF association networks’ have properties that are distinct from scale-free networks. First, a majority of the nodes do not have a preference for the number of neighbours, but instead node degrees can take a wide range of values (Supplementary Figure S3A). In other words, its distribution does not follow the power law as often seen in a scale-free network. Second, the average clustering coefficient for all nodes in the network does not follow a particular pattern and, therefore, suggests that the graph connectivity is not influenced by the number of neighbouring nodes (Supplementary Figure S3B). Third, nodes tend to be connected to other nodes of similar degree unlike GRNs where hubs tend to avoid each other (Supplementary Figure S3C). Taken together, these features indicate that a ‘TF association network’ can capture aspects of the combinatorial nature of TF regulatory interaction that are missed by correlation coefficient measures. Of note, the organization of TFs in this network shows a strong arrangement according to cell type rather than by TF (i.e. nodes of the same colour are grouped together), indicating that different TFs in the same cell type are more similar to each other than the same TFs in different cell types. An exception to this pattern is the observation that the *CTCF* TF forms a cluster on its own (bottom right subnetwork of Figure 3), consistent with the cell-type invariant function of CTCF [6, 27, 28]. Furthermore, the network also shows the strong dependence of *CTCF* on the cohesin complex (which includes Rad21 and Smc3) as reported previously [29].

We also found that TFs naturally organize into small clusters (Supplementary Figure S4), thereby emphasizing groups of similar TFs in a much clearer visualization than a heatmap of correlation coefficients. The network also provides a useful visualization for known interactions between seven TFs (*Erg, Fli1, Lmo2, Gata2, Tal1, Lyl1* and *Runx1*) in haematopoietic stem/progenitor cells (HSPC) [7] as shown by the subnetwork on the top right of Figure 3. To gain more mechanistic insights from this network, we annotated the edges in the network with protein–protein interaction data obtained from the STRING database [22]. We chose this data set as the gold standard because it is one of the most comprehensive databases of protein–protein interaction that is derived from multiple primary databases, has a large coverage of different proteins and consists of physical and functional interactions that are experimentally verified or computationally predicted. As this database contains known as well as predicted protein–protein interactions and includes both physical and functional associations, we decided to focus on high-confidence and physical interactions. Cooperative regulation by TF pairs is commonly associated with direct protein–protein interaction. As edges in our network represent likely direct interactions between two proteins that are commonly co-localized across the genome, we would expect a substantial proportion of these edges to be supported by evidence from the STRING protein–protein interaction database. In total, 222 edges were identified as high-confidence links in STRING and, of these, 46 edges are supported by evidence of physical interactions. However, evidence for interaction is not likely to be associated with a stronger partial correlation (GGM and graphical lasso) or normalized importance score (linear regression) of the edges in our network (Supplementary Figure S5). In Figure 3, thick grey edges represent high-confidence functional protein–protein interactions, whereas thick black edges indicate reported evidence of physical interactions between the proteins. For example, *LMO2* and *Gata2* as well as *Fli1* and *Runx1* have been shown to physically interact with each other (top right). Other examples of subnetworks show the strong dependence between mast cell TFs (middle right) [6] and the combination of three TFs—*Stat5*, *Irf4* and *Batf*—to be important for T-cell development (bottom left) [30, 31]. Overall, edges in our network represented known protein–protein interactions, but many edges are not supported by data from STRING. This is most likely because the STRING database has not included all protein–protein interactions published to date. It is also formally possible that a TF may influence DNA binding of another TF without the need for direct protein–protein interaction. Compared with the correlation method, however, our network is better at recovering known protein–protein interaction. We generated a correlation graph with similar number of edges as our ‘TF association network’ (1089 edges with correlation coefficient ≥ 0.55) and compared the number of known STRING records that were identified by both networks. Although both networks have similar precision rate (correlation graph—15%, TF association network—18%), the ‘TF association network’ had a better recall rate (correlation graph—14%, TF association network—31%) (Table 1). Moreover, the ‘TF association network’ had the highest precision rate (18%) compared with using any of the individual network inference methods alone. In other words, the fraction of network edges that are true protein–protein interactions is highest when using the consensus network approach. In terms of recall rate, however, the Bayesian network and GGM achieved >45% recall, while our ‘TF association network’ appeared to be limited by the interactions identified by the graphical lasso method (31%). As stated above, the graphical lasso method identified more unique edges that are nonoverlapping with the other methods (Supplementary Figure S1). Furthermore, many of the proteins in the graphical lasso method are not found in the STRING data set and, in total, 30 proteins that are in our data set have no evidence for protein–protein interaction in the STRING database. This suggests that many novel protein–protein interactions that control blood cell development are yet to be discovered.

**Table 1:** Performance comparison between the ‘TF association network’, the individual network inference algorithms and the correlation graph

Network inference method	TP	FP	TN	FN	Precision (%)	Recall (%)
Correlation graph	14	78	1366	82	15.22	14.58
GGM	44	248	1196	52	15.06	45.83
Graphical lasso	30	166	1278	66	15.31	31.25
Linear regression	35	252	1192	61	12.19	36.45
Bayesian network	47	268	1176	49	14.92	48.95
TF association network	30	131	1313	66	18.63	31.25

*Note*: Prediction rates (precision and recall) refer to the percentages of STRING protein–protein interactions identified by each network inference method. TP, true positive, FP, false positive, TN, true negative, FN, false negative.

**Figure 4:**
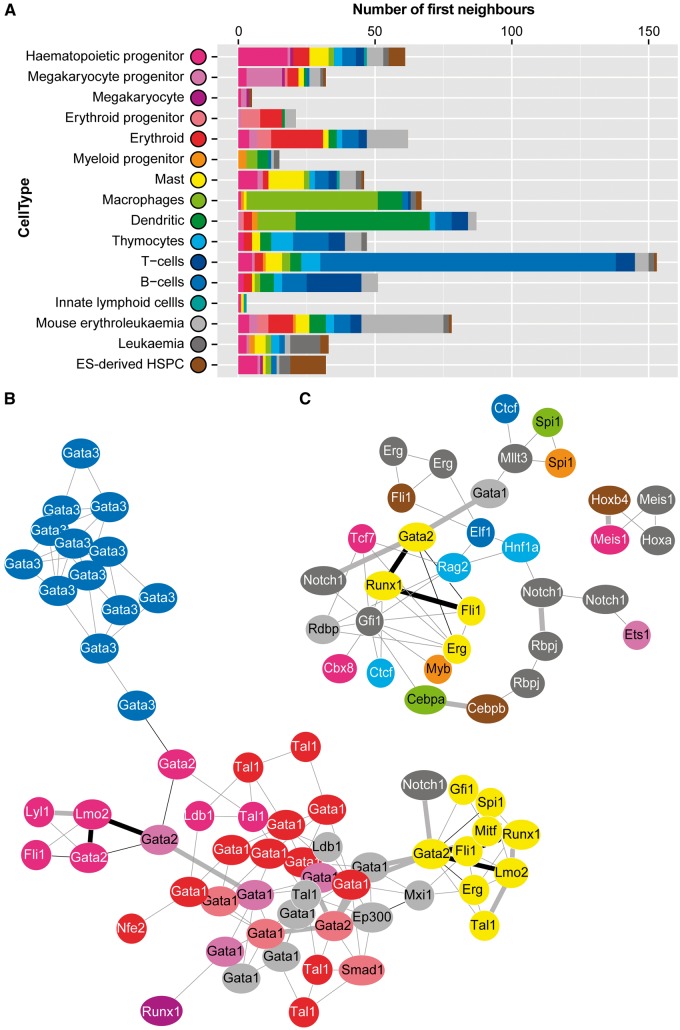
Analysis of TF association network. (**A**) First neighbour analysis. Bar chart shows the number of samples, which are first neighbour nodes and the cell type it belongs to. Barchart was plotted in R using the *ggplot2* package [32]. (**B**) Subnetwork of all GATA ChIP-seq samples and its closest neighbour nodes. Nodes are coloured by the cell type they belong to (Figure 4A). (**C**) Subnetwork of all leukaemia ChIP-seq samples and its closest neighbour nodes. Nodes are coloured by the cell type they belong to (Figure 4A).

Having shown that the ‘TF association network’ can illustrate conditional dependence relationships between ChIP-seq samples, we performed further network analysis to examine specific features of cell-type-specific TF binding. For each node in the network, we obtained all its first-neighbour nodes as well as the cell type each neighbour belongs to. By examining the first neighbours of each node in the network, we discovered that not only do TFs in the same cell type have similar binding but these TFs are more likely to associate with other TFs from a closely related haematopoietic cell type within the same lineage (Figure 4A). Moreover, HSPC derived from embryonic stem cells are closely related to haematopoietic progenitor samples, while samples from mouse erythroleukaemia and leukaemia are less biased to a subgroup of cell types, but instead show similarity to TF binding across all cell types. Figure 4B shows a subnetwork obtained by extracting only GATA TFs from the ‘TF association network’. In this subnetwork, we observed the utilization of specific GATA factors in distinct blood lineages—*Gata1* and *Gata2* in the myeloid lineage and *Gata3* in T cells, and *Gata3* TFs are independent of the binding of myeloid *Gata1* and *Gata2* TFs.

The study of TFs under diseased conditions is increasingly important for gaining mechanistic insights to transcriptional regulation during disease progression. It is also well known that genes encoding TFs account for many of the translocations observed in leukaemia patients. In this study, ChIP-seq samples from leukaemia studies (i.e. acute myeloid leukaemia, T-cell acute lymphoblastic leukaemia and the MLL–AF9 translocation) were incorporated into the ‘TF association network’ together with other samples of normal cell types. To investigate the TF-binding relationship between these two conditions, we plotted a subnetwork (Figure 4C) to display all the TFs from leukaemia studies together with their closest neighbour. Of note, this subnetwork shows the similarity of TFs in leukaemia to many progenitor samples (haematopoietic progenitor, megakaryocyte progenitor and myeloid progenitor). Although one might have expected that leukemic TFs would resemble the binding of the same TF in one of the normal cell types, we found that the genome-wide binding of these TFs can in fact change dramatically during cancer progression to resemble the binding of a completely different TF.

## Discussion

As a result of the advancements in ChIP-seq technology and its widespread uptake by individual laboratories as well as large-scale consortium efforts, it is now possible to integrate publicly available ChIP-seq experiments to perform large-scale quantitative and qualitative meta-analysis of genome-wide TF-binding profiles. For example, various bioinformatics approaches have been used to uncover protein–protein interactions from a collection of ChIP-seq data to gain a global understanding of combinatorial TF binding and interaction networks in the regulation of cell fate decisions [33, 34]. In a recent study, our group also showed that the integration of disparate public TF ChIP-seq experiments of blood-related samples can form a coherent picture of constrained sequence-specific TF pair interaction with the DNA [35]. Here, we demonstrate that correlation coefficient as a measure for TF relationships has limitations given the large numbers of cell-type-specific binding regions in a compendium of samples coming from a broad range of haematopoietic cell types. We further showed that graphical modelling theory has the added advantage of joint network modelling, provides the fundamentals for constructing conditional dependence graphs to detect likely direct dependence between TFs and the resulting ‘TF association network’ provides an intuitive data visualization technique for understanding regulatory relationships between TF ChIP-seq samples. The notion of conditional independence enabled the discovery of ‘direct’ TF interactions that are consistent with our knowledge of TF function because (i) TFs bind to the DNA with differing affinity, (ii) different TFs can compete for the same set of binding sites and (iii) TFs cooperate to form synergistic interactions. In contrast, correlation networks applied to this type of data would only uncover the marginal dependence structure among TFs. Using four different algorithms to obtain conditional dependence measures of TF binding on all regulatory elements bound in haematopoietic cells, we were able to (i) construct a ‘TF association network’ that is suggestive of the causal structure underlying the observed dependencies, (ii) visualize the relationships between ChIP-seq samples of not only normal haematopoietic cell types but also leukemic cells, (iii) uncover likely mechanisms of TF binding including combinatorial regulation, cell-type-specific regulators and binding site reorganization during haematopoietic differentiation and (iv) identify cell-type-specific as well as shared TFs.

By treating the ‘TF association network’ inference algorithms as a sparse feature selection problem, the most important edges in the network can be discovered. Controlling the network sparsity, therefore, will produce a network where few TFs interact with each other. Although the algorithms used in this study were initially developed for high-dimensional data (i.e. small *n*, large *p*), they provide quick computation of the conditional dependence measures for TF-binding data (i.e. very large *n*, large *p*) and, more importantly, the simultaneous selection of the most important TF variables directly impacting on each TF. In this study, we did not use any tree-based network inference algorithms (such as random forest) because the computation time was too long for several hundred samples, but would have been feasible for a smaller data set. Edges in the network were supported by experimental evidence of known protein–protein interactions in the STRING database. Many edges that were not supported by STRING are in fact known interactions that have not been added to the database. For example, *Gata2/Fli1/Tal1* is an important regulatory circuit, and *Ldb1* is known to interact with *Tal1* as part of a specific complex involving *Lmo2, Tcf3, Gata1, Tal1* and *Ldb1* during haematopoietic development (Supplementary Figure S6) [36, 37]. Consistent with observations from several independent studies, we found that TFs undergo substantial binding site reorganization during development [1, 6, 9, 38] and are differentially recruited in different blood cell types [5, 39]. However, it is important to note that direct interactions in a ‘TF association network’ may or may not be associated with cell-type-specific gene expression or the hierarchy of TF recruitment, although these are known to have an important influence on cell fates [40, 41]. To do this, we suggest that additional data such as gene expression are incorporated into the network analysis. Moreover, the algorithms used in this study only assume an average binding effect between two TFs, but make no inference about the different modes of interaction (activation, repression or both).

Unlike GRNs where highly connected nodes (hubs) are surrounded by low-degree nodes and hubs tend to avoid each other, nodes in our TF association network have a preference for attaching to other nodes of similar degree, which in itself is highly suggestive of combinatorial TF binding. Interestingly, the network not only provides a global view of TFs that are important for haematopoiesis including a global view of lineage-specific factors but also the current state of knowledge of known TFs that are important for the function of haematopoietic cell types. Using this network, investigators may choose to prioritize certain experiments or define a hypothesis for further testing. The nearest neighbour analysis, in contrast, suggests potential factors for TF-mediated cellular reprogramming and may provide an indication of the shortest path in which somatic cell reprogramming of cell-type-specific enhancers may take place. The overexpression of a set of defined factors whose binding is most similar and possibly acting as co-factors is likely to be more efficient in remodelling the genome to a pluripotent state than the overexpression of factors whose binding are different. Our network also allowed comparison of TFs in normal cell types with leukemic samples and showed the relationships between TF binding and the leukaemia phenotype. In particular, TFs in leukemic samples undergo substantial binding reorganization and are similar to many factors in progenitor cell types, although similarity to some mature cell types was also observed. Our network also uncovered potential co-factors that may be recruited to these new binding sites in leukaemia. This is consistent with observations where dysregulated TFs in leukaemia can lead to a differentiation block through changes in TF-binding ability and impaired recruitment of co-factors [42].

Compared with recent studies using large-scale ChIP-seq data sets, our method provides a more intuitive comparison of all samples from multiple cell types and how they are related to each other. Moreover, it serves as a framework for generating new hypothesis on potential TF partners. Self-organizing maps, non-negative matrix factorization and probabilistic itemset mining are among some of the computational methodologies that have been used to infer TF co-localization patterns from TF-binding profiles [34, 43, 44]. Important protein complexes are then selected from commonly occurring co-localization patterns if they influence cell-type-specific gene expression. Graphical models, on the other hand, have been applied to ChIP-seq data sets in *Drosophila melanogaster*, but in a much smaller data set [45], while Gaussian graphical models have been shown to be a stable solution for uncovering reactions in a metabolic pathway [46]. Our method differs from these studies because a ‘TF association’ network facilitates the direct comparison of TFs in multiple cell types within a single visualization, as the joint probability distribution was calculated on all samples. Moreover, the uniformly processed data in CODEX facilitate the comparison of data from different laboratories. Using standard techniques to analyse our network, we were able to also uncover the cell-type-specific and shared relationships between TFs across haematopoietic development. Nodes that do not seem to cluster with the cell type or TF they belong to can also serve as a proxy for a missing TF in the input data set. A recent study took a similar approach to generate a graphical model for ENCODE TF and histone modification ChIP-seq data and showed that conditional dependence measures can easily scale up to large data sets as well as discover known protein–protein interactions [47]. Indeed, the interplay between TF binding and epigenetic state is crucial for determining cell fate, and therefore, may influence the topology of the TF association network. Cell-type-specific epigenetic profiles, however, have been shown to be largely driven by the expression of lineage-restricted TFs, and the relationship between epigenetic state and TF binding is tightly linked and mutually impact each other [4, 41, 48, 49]. Instead of directly incorporating chromatin data into our model, however, we believe a better approach would be to create a directional network with hierarchical organization of chromatin and TF influence. In such a network, the conditional dependence relationship is imposed by the hierarchy, and the combined chromatin and TF model would, therefore, consist of directional edges with causal interpretation between epigenetic states and TF binding.

Taken together, the TF association network is a flexible form of graphical representation and may be adapted further to suit different analysis requirements. For example, the conditional dependence measures may be calculated from peak height values instead, or weighted edges may be inferred from the partial correlation coefficient values. DNA methylation, epigenetic mechanisms and DNA accessibility are all known to influence TF binding [50–52], but have not been investigated here. Future integration of such data sets (e.g. MNAse-seq, DNAse-seq, FAIRE-seq, ATAC-seq, etc.) will provide greater insight into how these additional layers of gene regulation influence the conditional dependence structure of a ‘TF association network’.

**Key Points**

- Commonly used methods for the comparison of multiple TF-binding maps are not adapted to visualizing transcriptional regulation across diverse developmental stages.
- Here, we report an alternative visualization approach using graphical modelling theory that not only discovers functionally relevant regulatory relationships but illustrates TF influences of a diverse set of TFs across the spectrum of mouse blood cell development.
- The notion of conditional independence and simultaneous variable selection of TF influences represents an intuitive approach to discovering ‘direct’ dependence between TFs.
- The resulting network illustrates key features of TF function including combinatorial TF regulation, cell-type-specific regulators, protein–protein interaction and TF-binding reorganization in leukemic cells.

## Supplementary data

Supplementary data are available online at http://bib.oxfordjournals.org/.

## Funding

Bloodwise, the Biotechnology and Biological Sciences Research Council, the Leukaemia and Lymphoma Society, Cancer Research UK, the National Institute for Health Research Cambridge Biomedical Research Centre, and the Wellcome Trust and MRC Cambridge Institute for Medical Research and Wellcome Trust—Medical Research Council Cambridge Stem Cell Institute; Yousef Jameel scholarship awarded by the Cambridge Commonwealth, European and International Trust (to F.S.L.N.).

## Supplementary Material

Supplementary Data

## Notes

**Felicia Ng** is a postdoctoral researcher at the Department of Haematology, Wellcome Trust and MRC Cambridge Stem Cell Institute and Cambridge Institute for Medical Research.

## Notes

**David Ruau** is a postdoctoral researcher at the Department of Haematology, Wellcome Trust and MRC Cambridge Stem Cell Institute and Cambridge Institute for Medical Research.

## Notes

**Lorenz Wernisch** is a Programme Leader of Statistical Genomics at the Medical Research Council Biostatistics Unit.

## Notes

**Berthold Göttgens** is a Principal Investigator and Professor of Molecular Haematology at the Department of Haematology, Wellcome Trust and MRC Cambridge Stem Cell Institute and Cambridge Institute for Medical Research.
