# Supplementary material for: A graphical model approach visualizes regulatory relationships between genome-wide transcription factor binding profiles
Source: Brief Bioinform. 2016 Oct 25;19(1):162–73. doi: 10.1093/bib/bbw102 (PMC5496675; doi:10.1093/bib/bbw102)
Supplement: Supplementary Data [file bbw102_supplementary_materials.pdf]

A graphical model approach visualizes regulatory relationships between genome-wide transcription factor binding profiles

Felicia SL Ng[1], David Ruau[1], Lorenz Wernisch[2], Berthold Gottgens[1]

[1] Department of Haematology, Wellcome Trust and MRC Cambridge Stem Cell Institute & Cambridge Institute for Medical Research, University of Cambridge, Cambridge, UK. [2] MRC Biostatistics Unit, Cambridge Institute of Public Health, Cambridge, UK

SUPPLEMENTARY MATERIALS

Supplementary Methods

Network inference algorithms for constructing a TF association network

For a set of p ChIP-seq samples, a TF association network $\mathcal{G} = (V, E)$ can be constructed to illustrate the similarities in genome-wide DNA binding between samples. To do this, we used the multi-sample TF binding profile, X, as input data to determine causal relationships amongst all possible pairs of TFs (except with itself), and further assume that each ChIP-seq experiment correspond to one variable in the model regardless of multiple experiments immuno-precipitating the same TF in the same cell type. Consequently, this network is a direct dependence graph for the set of TFs $T = [1, p]$ where each $t_j \in T, j = 1, \dots, p$ is one ChIP-seq experiment (note that Tconsists of the non-unique set of all TFs). This network contains the set of vertices (i.e. nodes) $V = \{v_1, v_2, \dots, v_p\}$ and the set of undirected vertex pairs (i.e. edges) $E \subset \{(v_j, v_{j'}), j, j' = 1, \dots, p, j \neq j'\}$. In a TF association network,

a node corresponds to one TF $t_j \in T$ while an edge corresponds to the direct dependence between the bindings of a pair of TFs.

When analyzing multiple ChIP-seq samples, many studies infer TF binding similarity by using correlation or mutual information measures. However, a high correlation coefficient or mutual information between the bindings of any two TFs may suggest different scenarios: a 'direct' interaction, an 'indirect' interaction or regulation by a common TF. Therefore, in the context of a 'TF association network', only 'direct' interactions are relevant as these correspond to edges between nodes (TFs) in the resulting graph and the main purpose of a network inference algorithm is to discover the set E of direct interactions or significant links between nodes (TFs) from the input data. Crucially, the algorithms should be able to distinguish 'direct' interactions from 'indirect' interactions (e.g. two highly correlated TFs that are similarly regulated by another TF). Instead, the notion of conditional independence commonly applied in gene regulatory network algorithms provides a useful way to determine the set of 'true' interactions. To achieve this, we utilized various network inference algorithms (see below) to obtain a matrix $M$of size $p \times p$ where each entry in the matrix is a measure of direct dependence between t_j and $t_{j'}$. To select important or significant edges, a threshold (differs for each algorithm) is applied to the matrix $M$to obtain an adjacency matrix and discover 'true' interaction edges as follows - matrix elements that pass the threshold are given a score of '1' and '0' otherwise. This denotes the presence and absence of an edge, respectively. The adjacency matrix defines which edges should be plotted in the resulting network and all edges are treated as un-weighted. Only off-diagonal elements in the upper or lower matrix are used for constructing the network as these correspond to an undirected graph with no self-regulation. In other words, we do not assume any directionality or hierarchy in the TF interactions and TFs do not regulate itself.

In this study, four types of network inference algorithms were employed to obtain the matrix M of direct dependence measures. They were chosen based on their known performance in gene

regulatory network inference, computational efficiency and coverage across different classes of graphical modeling theory.

(1) Gaussian Graphical Model (GGM) – This method was first developed to model the gene association structures from microarray data [1] and produces an undirected graph. We adapted this method to generate a 'TF association network' as follows. Given a TF binding matrix, X, as input data, conditional dependence between TFs t_j and $t_{j'}$ is given by the partial correlation of X_j and $X_{j'}$:

$$\mathbb{C}\mathrm{or}\left(X_j, X_{j'} \middle| (X_k)_{k \neq j,j'}\right) = \rho\left(X_j, X_{j'} | X_k\right) \qquad \forall\, j, j', k \in P \tag{1}$$

Standard graphical modeling theory shows that the partial correlations may be calculated by inversion of the covariance matrix. However, for large datasets, conventional approaches for obtaining the covariance matrix (i.e. the unbiased empirical covariance matrix and the maximum likelihood estimate) are known to be statistically inefficient and ill-conditioned. Hence, Schäfer and Strimmer proposed the use of shrinkage estimators of the covariance matrix [2] and showed that the shrinkage approach provides a more accurate, well-conditioned and always positive-definite covariance matrix. Moreover, it is computationally more efficient compared to conventional approaches. In this study, we used the *GeneNet* package in R [3] to compute a reliable estimate of the partial correlation matrix for all pairs of TFs. Statistical significance is then assigned to the edges in the GGM network by fitting a mixture model to the observed partial correlations and computing the p-value and posterior probabilities for each edge. Significant non-zero partial correlations are obtained using the empirical Bayes local false discovery rate (fdr) statistic where edges less than 1e-10 are considered significant. In total, 366 nodes and 2076 undirected and non-self-regulating edges were identified in the GGM network.

(2) Graphical lasso – Graphical lasso [4] is another approach to estimate the covariance matrix as part of learning the structure of an undirected graphical model. Under this framework, the inverse covariance matrix is assumed to be sparse and the $\ell_1$regularization parameter controls the number of zeros in the matrix. Any inverse covariance matrix element with the value of zero implies that the corresponding variables X_j and X_j, are conditionally independent, given the rest of the dataset. The simultaneous estimation and selection of the partial correlations, therefore, is an intuitive way of introducing sparsity in the network.

The TF association network in this study was generated by calculating a shrunken partial correlation matrix using the *glasso* R package [5]. We set the $\ell_1$regularization parameter ('rho' option in the 'glasso' function) to 0.01 and considered an edge to be significant if the partial correlation value is non-zero. For any pair of TFs, the partial correlation value is taken as the average between elements in the corresponding upper and lower triangular matrix values. Any non-zero self-regulating edges were removed. We obtained 196 nodes and 2033 undirected edges in the network generated using this algorithm.

(3) Linear regression – Linear regression-based approaches treat each variable or node in the network as a linear combination of all the other variables in the input dataset. For example, we can predict the binding of TF t_j as a function of the binding of all other TFs in the dataset and to construct a network for all TFs in the dataset simply involve a series of linear regression equations. By identifying non-zero regression coefficients, we can discover which TFs are directly interacting with t_j and thereby assign an edge. In this scenario, network inference is a feature selection problem performed node by node to identify significant and non-zero coefficients which corresponds to direct dependence relationships. TIGRESS [6] is one such network inference algorithm that combines the multivariate feature selection method known as least angle regression (LARS) [7] with stability

selection [8] to achieve this. Given a TF binding matrix, X, as input data, we used TIGRESS to model each TF $t_j$as a function of all other TFs $t_{j'}$ $(j' \neq j)$ in the input dataset by using the linear regression model:

$$X_j = \sum_{j'=1,j'\neq j}^{p} \beta_{jj'} X_{j'} + \epsilon_{j'} \tag{2}$$

For each TF, LARS selects the set of predictor variables $X_{j'}$,that best explains the binding of that TF so that indirect interactions are omitted. However, LARS is known to be sensitive and unstable for datasets where variables are highly correlated. Therefore, stability selection is employed to run LARS many times (samples and variables resampled at each run) to compute the frequency with which each variable were selected by LARS. This is then used to calculate the normalized score for each TF, $s_{j'}$ $\forall j' \neq j$, as a measure of direct dependence on the binding of TF t_j.

We used the TIGRESS software (MATLAB code obtained from (http://cbio.ensmp.fr/~ahaury/svn/dream5/html/index.html) to calculate the direct dependence matrix for all TFs in the input dataset. For each TF t_j, the normalized score for the dependence of TF $t_{j'}$ is taken as the average between elements in the corresponding upper and lower triangular matrix values. The adjacency matrix was defined using a threshold of $s_{j'} \geq 0.07$ and the resulting TF association network contained 367 nodes and 2123 edges.

(4) Bayesian Network – We estimated a Bayesian Network (BN) structure that display the conditional independence relationships of a set ChIP-seq samples based on the *Markov condition*, which states that each variable in the model (node) is conditionally independent of the set of all its predecessors in the graph, given the value of its parents. The structure of a BN in a 'TF association network', therefore, represents the factorization of the joint probability of all ChIP-seq samples:

$$P[X_1, X_2, \ldots, X_p] = \prod_{j=1}^{p} P[X_j | pa_j] \quad (3)$$

where pa_j is the set of parents of node X_j.

Unlike the three methods described earlier, the conditional independence tests in a BN are estimated concurrently as part of the network structure learning procedure. The resulting network takes the form of a directed acyclic graph (DAG) where directed edges between two nodes indicate the direction of causal influence. However, we do not enforce the notion of causality in our networks since the directions only represent associations but not genuine causal relationships. Instead, we interpret all edges as undirected.

Using the *bnlearn* R package [9], the conditional independence structure of the input data was learned using the score-based hill climbing algorithm. The adjacency matrix was computed by taking into account only off-diagonal entries in the upper/lower matrix and treating all edges as undirected. In total, there were 367 nodes and 2013 edges in the network.

Consensus network – To obtain a consensus network for further analysis, we constructed a new network based on the edges that were discovered in any 3 or more algorithms and the resulting graph consists of 362 nodes and 1182 edges. Cytoscape version 3.2.1 was used to visualize the network [10]. Source code for the generating the consensus network can be downloaded at (https://bitbucket.org/feliciang/publication_tf_association_network).

References

1. Kishino H, Waddell PJ. Correspondence analysis of genes and tissue types and finding genetic links from microarray data, Genome Inform Ser Workshop Genome Inform 2000;11:83-95.

2. Schafer J, Strimmer K. A shrinkage approach to large-scale covariance matrix estimation and implications for functional genomics, Stat Appl Genet Mol Biol 2005;4:Article32.

3. Schaefer J, Opgen-Rhein R, Strimmer K. GeneNet: Modeling and Inferring Gene Networks. 2015.

4. Friedman J, Hastie T, Tibshirani R. Sparse inverse covariance estimation with the graphical lasso, Biostatistics 2008;9:432-441.

5. Friedman J, Hastie T, Tibshirani R. glasso: Graphical lasso- estimation of Gaussian graphical models. 2014.

6. Haury AC, Mordelet F, Vera-Licona P et al. TIGRESS: Trustful Inference of Gene REgulation using Stability Selection, BMC Syst Biol 2012;6:145.

7. Efron B, Hastie T, Johnstone I et al. Least angle regression 2004:407-499.

8. Meinshausen N, Bühlmann P. Stability selection, Journal of the Royal Statistical Society: Series B (Statistical Methodology) 2010;72:417-473.

9. Scutari M. Learning Bayesian Networks with the bnlearn R Package, Journal of Statistical Software 2010;35:1-22.

10. Shannon P, Markiel A, Ozier O et al. Cytoscape: a software environment for integrated models of biomolecular interaction networks, Genome Res 2003;13:2498-2504.

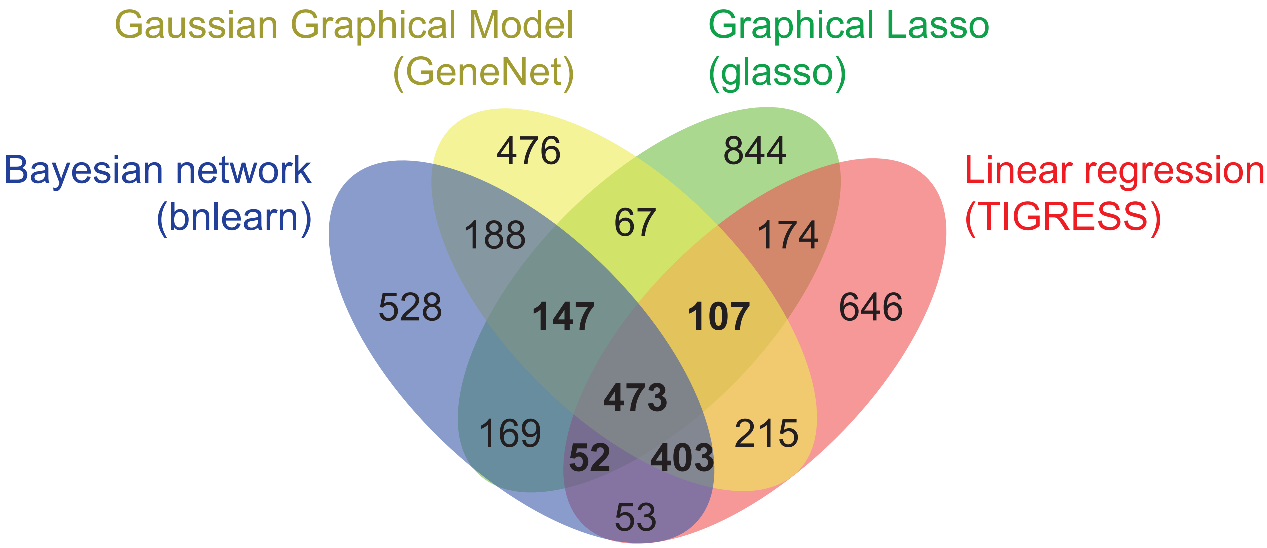

Supplementary Figure 1 Venn diagram shows the extent of overlap of edges discovered by each algorithm. Numbers in bold indicate the number of edges discovered by at least 3 algorithms.

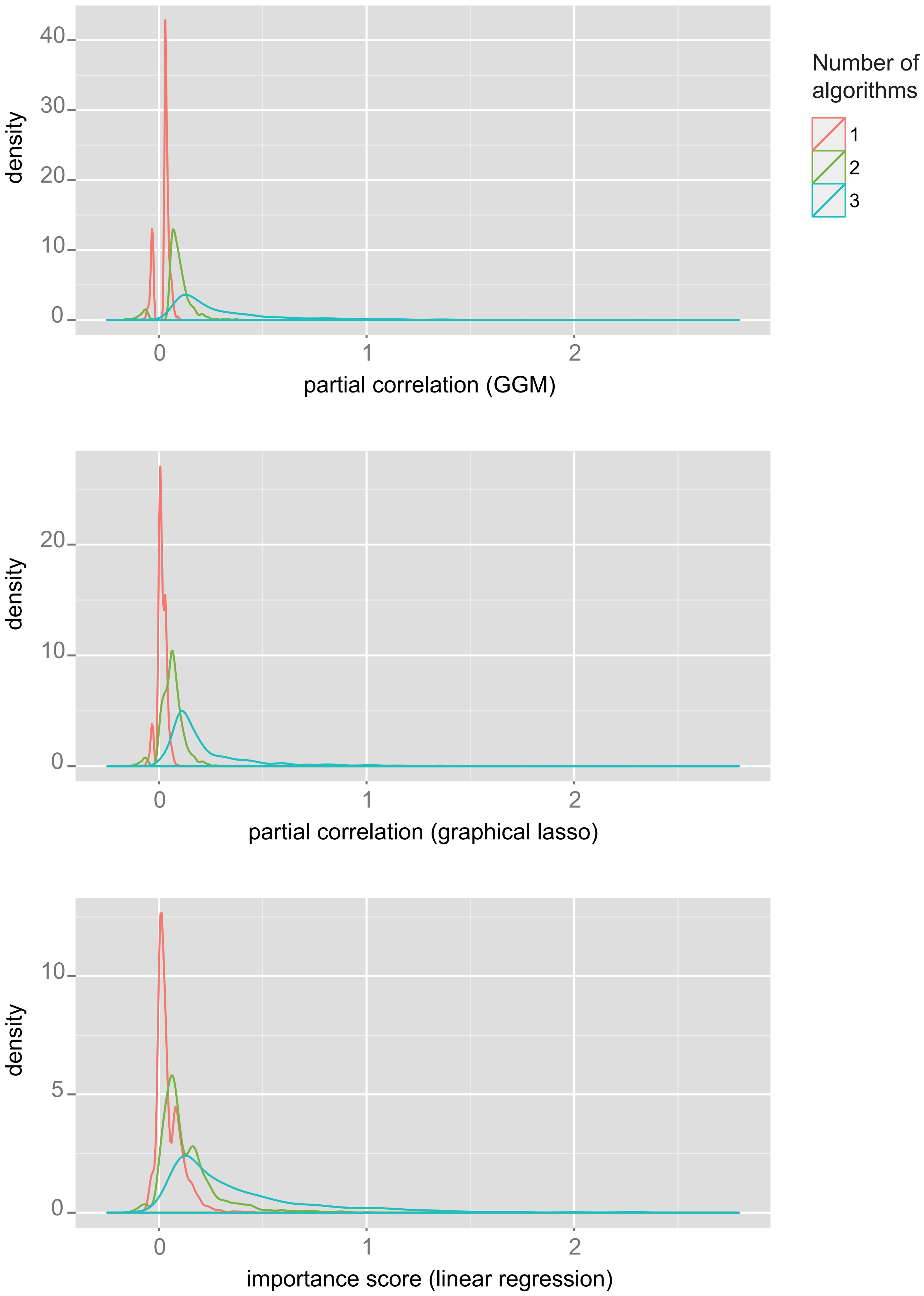

Supplementary Figure 2 Distribution of direct dependence measures (i.e. partial correlation or importance scores) estimated by the GGM, graphical lasso and linear regression methods. Density plots in red, green and blue lines represent edges discovered by at least one, two, and three algorithms respectively.

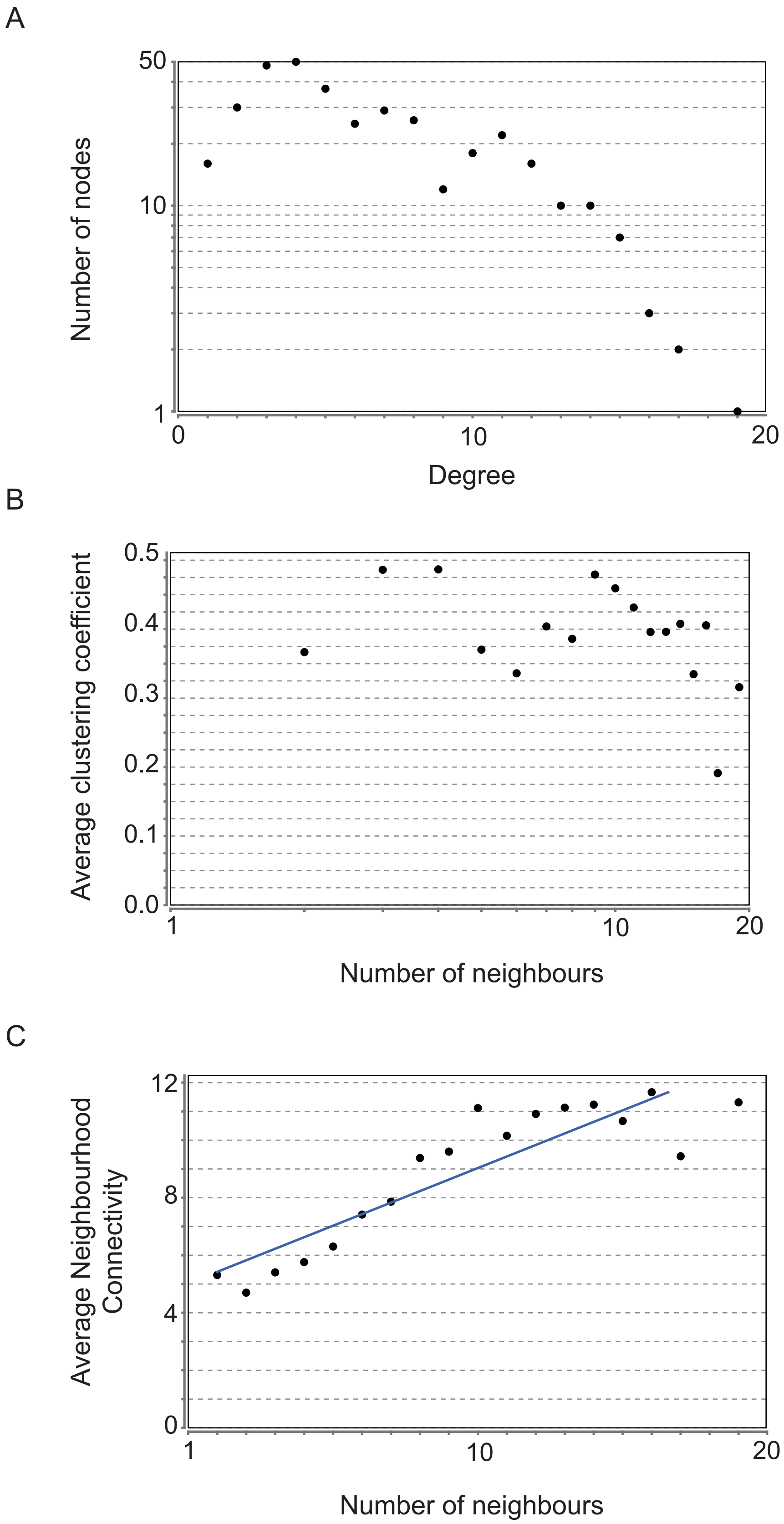

Supplementary Figure 3 Network statistics for the TF association network (Figure 3). Analysis was performed in Cytoscape. (A) Distribution of node degrees for all nodes in the network. (B) Average clustering coefficient. Scatter plot shows the number of connected pairs between all neighbours. (C) Average neighbourhood connectivity. Scatter plot shows the extents to which nodes are connected to other nodes of similar degree (number of neighbours vs average neighbourhood connectivity) and the blue line is a fitted linear regression line.

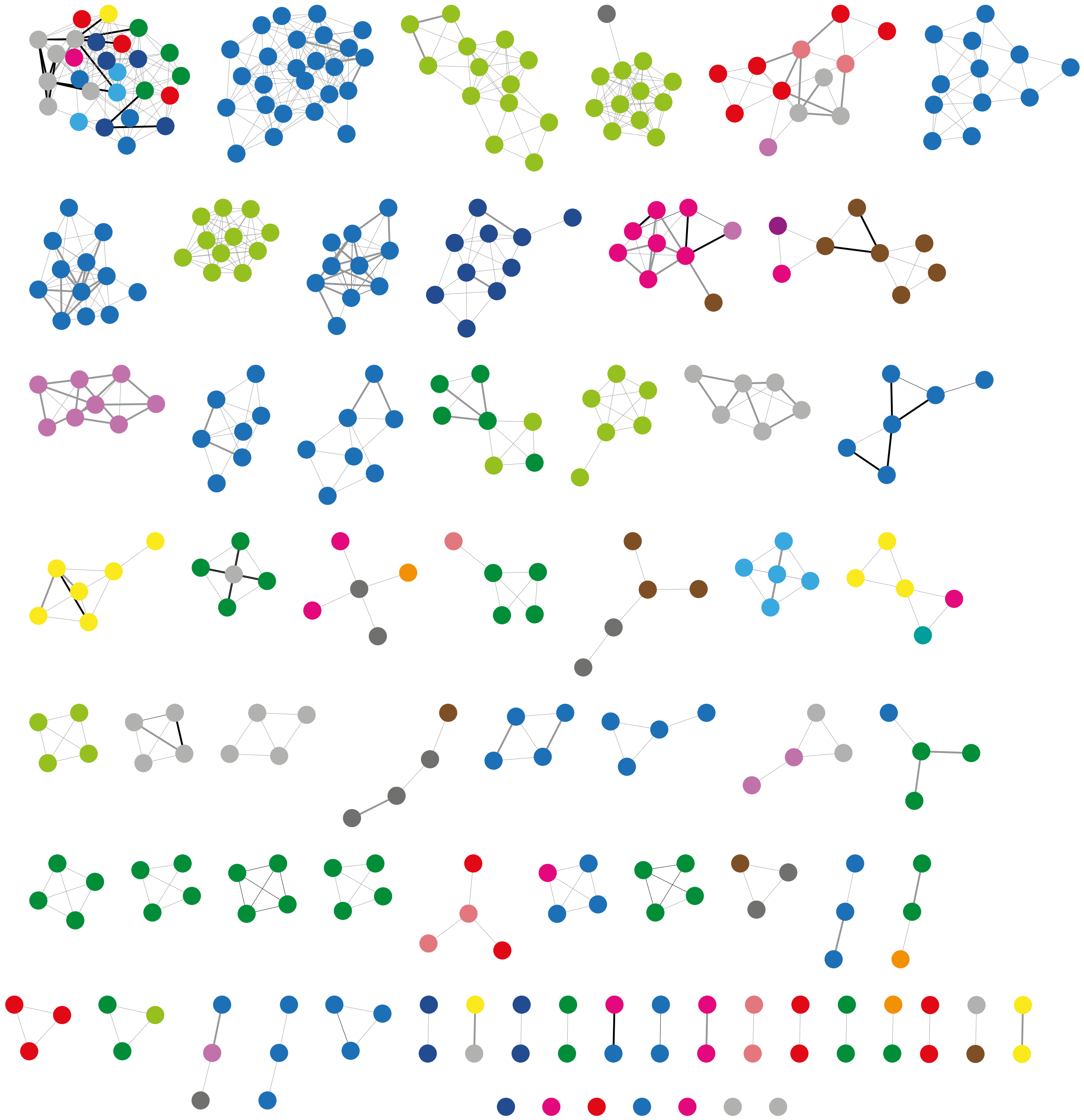

Supplementary Figure 4 Network clustering of ChIP-seq samples performed by the Markov Cluster (MCL) algorithm in Cytoscape. Each node is coloured by the cell type it belongs to.

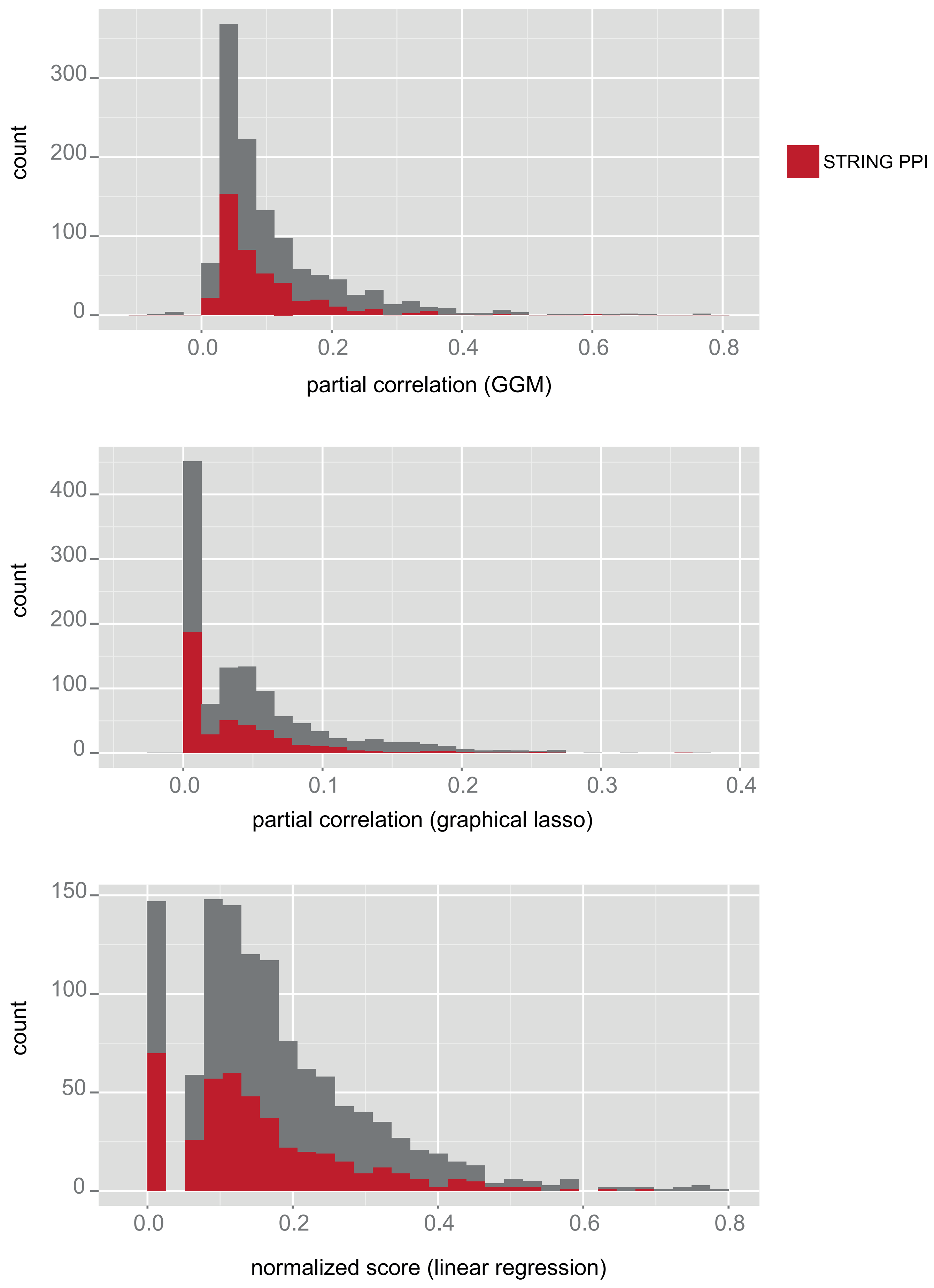

Supplementary Figure 5 Distribution of direct dependence measures (i.e. partial correlation or importance scores) estimated by the GGM, graphical lasso and linear regression methods. Histrogram barchart coloured in red represent edges whose protein-protein interactions are supported by data in the STRING database.

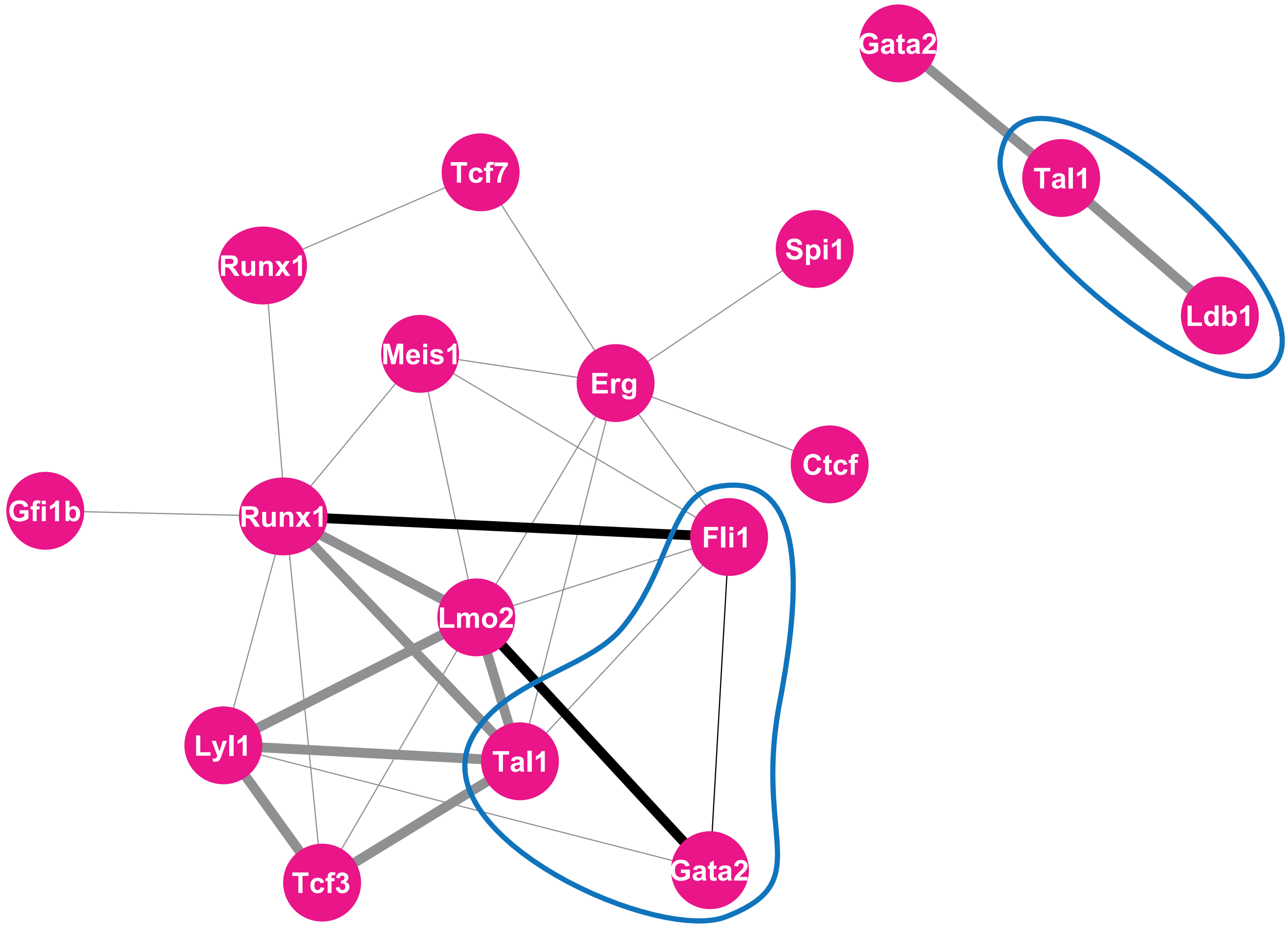

Supplementary Figure 6 Subnetwork for haematopoietic progenitor samples only. Examples of known protein-protein interactions in haematopoietic progenitors that are not in the STRING database are highlighted in blue.

Supplementary Table 1

Details of the 367 ChIP-seq samples

transcription_factor	gene_ID	GSE	GSM	cell_type
Ctcf	13018	GSE21978	GSM546526	B-Cells
Ctcf	13018	GSE26257	GSM644975	B-Cells
Ctcf	13018	GSE41743	GSM1023420	B-Cells
Ebf1	13591	GSE19971	GSM499030	B-Cells
Ebf1	13591	GSE35857	GSM876622-GSM876623	B-Cells
Ebf1	13591	GSE35857	GSM876624-GSM876625	B-Cells
Foxo1	56458	GSE21978	GSM546525	B-Cells
Ikzf1	22778	GSE38200	GSM936201-GSM936202	B-Cells
Ikzf1	22778	GSE38200	GSM936199-GSM936200	B-Cells
Ikzf1	22778	GSE38200	GSM1040573-GSM1040574	B-Cells
Irf4	16364	GSE39756	GSM978747	B-Cells
Irf4	16364	GSE39756	GSM978748	B-Cells
Med1	19014	GSE44288	GSM1038263	B-Cells
Pax5	18507	GSE38046	GSM932925-GSM932931	B-Cells
Pax5	18507	GSE38046	GSM932921-GSM932924	B-Cells
Pou2f2	18987	GSE21512	GSM537990	B-Cells
Rad21	19357	GSE26257	GSM644976	B-Cells
Spi1	20375	GSE21512	GSM537989	B-Cells
Spi1	20375	GSE21614	GSM539537-GSM539538	B-Cells
Smad3	17127	GSE21614	GSM539545	B-Cells
Batf	53314	GSE52773	GSM1288392	Dendritic
Ctcf	13018	GSE36099	GSM881052	Dendritic
Ctcf	13018	GSE36099	GSM881053	Dendritic
Ctcf	13018	GSE36099	GSM881054	Dendritic
Ctcf	13018	GSE36099	GSM881055	Dendritic
E2f1	13555	GSE36099	GSM881056	Dendritic
E2f1	13555	GSE36099	GSM881057	Dendritic
E2f1	13555	GSE36099	GSM881058	Dendritic
E2f1	13555	GSE36099	GSM881059	Dendritic
E2f4	104394	GSE36099	GSM881060	Dendritic
E2f4	104394	GSE36099	GSM881061	Dendritic
E2f4	104394	GSE36099	GSM881062	Dendritic
E2f4	104394	GSE36099	GSM881063	Dendritic
Egr1	13653	GSE36099	GSM881140	Dendritic
Egr1	13653	GSE36099	GSM881142	Dendritic
Egr2	13654	GSE36099	GSM881093	Dendritic
Ets2	23872	GSE36099	GSM881064	Dendritic
Ets2	23872	GSE36099	GSM881065	Dendritic
Ets2	23872	GSE36099	GSM881066	Dendritic
Ets2	23872	GSE36099	GSM881067	Dendritic
Hif1a	15251	GSE36099	GSM881068	Dendritic
Hif1a	15251	GSE36099	GSM881069	Dendritic
Hif1a	15251	GSE36099	GSM881070	Dendritic
Hif1a	15251	GSE36099	GSM881071	Dendritic
Irf1	16362	GSE36099	GSM881072	Dendritic
Irf1	16362	GSE36099	GSM881073	Dendritic
Irf1	16362	GSE36099	GSM881074	Dendritic
Irf1	16362	GSE36099	GSM881075	Dendritic
Irf8	15900	GSE53311	GSM1289235	Dendritic
Irf8	15900	GSE53311	GSM1289237	Dendritic
JunB	16477	GSE52773	GSM1288393	Dendritic
Maff	17133	GSE36099	GSM881095	Dendritic
Maff	17133	GSE36099	GSM881096	Dendritic
Maff	17133	GSE36099	GSM881097	Dendritic
Maff	17133	GSE36099	GSM881098	Dendritic
Rel	19696	GSE36099	GSM881132	Dendritic
Rel	19696	GSE36099	GSM881134	Dendritic
Rela	19697	GSE36099	GSM881111	Dendritic
Rela	19697	GSE36099	GSM881112	Dendritic
Rela	19697	GSE36099	GSM881113	Dendritic
Rela	19697	GSE36099	GSM881114	Dendritic
Relb	19698	GSE36099	GSM881110	Dendritic
Spi1	20375	GSE52773	GSM1288391	Dendritic
Stat3	20848	GSE27161	GSM671415	Dendritic
Stat3	20848	GSE27161	GSM671416	Dendritic
Stat3	20848	GSE36099	GSM881119	Dendritic
Stat3	20848	GSE36099	GSM881121	Dendritic
Stat5b	20851	GSE27161	GSM671418	Dendritic
Stat5b	20851	GSE27161	GSM671419	Dendritic

Cebpb	12608	GSE40235	GSM989017	EmbryonicStemCells
Fli1	14247	GSE40235	GSM989023	EmbryonicStemCells
Fli1	14247	GSE40235	GSM989015	EmbryonicStemCells
Fli1	14247	GSE40235	GSM989016	EmbryonicStemCells
Hoxb4	15412	GSE34014	GSM840466	EmbryonicStemCells
Hoxb4	15412	GSE34014	GSM840468	EmbryonicStemCells
Hoxb4	15412	GSE34014	GSM840470	EmbryonicStemCells
Ldb1	16825	GSE43044	GSM1055553	EmbryonicStemCells
Runx1	12394	GSE29515	GSM730630	EmbryonicStemCells
Runx1	12394	GSE40235	GSM989013	EmbryonicStemCells
Runx1	12394	GSE40235	GSM989014	EmbryonicStemCells
Tal1	21349	GSE40235	GSM989024	EmbryonicStemCells
Tal1	21349	GSE40235	GSM989018	EmbryonicStemCells
Tal1	21349	GSE40235	GSM989019	EmbryonicStemCells
Ctcf	13018	GSE36029	GSM923570	Erythroid
Ctcf	13018	GSE36029	GSM923571	Erythroid
Ctcf	13018	GSE47492	GSM1151145	Erythroid
Gata1	14460	GSE30142	GSM746581-GSM746582	Erythroid
Gata1	14460	GSE18164	GSM453997	Erythroid
Gata1	14460	GSE36029	GSM923572	Erythroid
Gata1	14460	GSE36029	GSM923575	Erythroid
Gata1	14460	ERA195911	ERX206185-ERX206187	Erythroid
Gata1	14460	ERA195911	ERX206186-ERX206189	Erythroid
Gata1	14460	GSE47492	GSM1151146	Erythroid
Klf1	16596	GSE48020	GSM1165493	Erythroid
Nfe2	18022	GSE47492	GSM1151147	Erythroid
Tal1	21349	GSE30142	GSM746571-GSM746572	Erythroid
Tal1	21349	GSE30142	GSM746555-GSM746556	Erythroid
Tal1	21349	GSE30142	GSM746583-GSM746584	Erythroid
Tal1	21349	GSE18720	GSM464634	Erythroid
Tal1	21349	GSE18720	GSM464636	Erythroid
Tal1	21349	GSE36029	GSM923576	Erythroid
Tal1	21349	GSE36029	GSM923579	Erythroid
Gata1	14460	GSE29193	GSM722390	ErythroidProgenitors
Gata1	14460	GSE35385	GSM867156-GSM867157	ErythroidProgenitors
Gata1	14460	GSE35385	GSM867158-GSM867160	ErythroidProgenitors
Gata2	14461	GSE29193	GSM722387	ErythroidProgenitors
Spi1	20375	GSE21953	GSM545888-GSM545891	ErythroidProgenitors
Spi1	20375	GSE21953	GSM545882-GSM545885	ErythroidProgenitors
Smad1	17125	GSE29193	GSM722388	ErythroidProgenitors
Smad1	17125	GSE29193	GSM722391	ErythroidProgenitors
Cbx7	52609	GSE36658	GSM898200	HaematopoieticProgenitors
Cbx8	30951	GSE36658	GSM898201	HaematopoieticProgenitors
Ctcf	13018	GSE48086	GSM1167572	HaematopoieticProgenitors
Erg	13876	NA	NA	HaematopoieticProgenitors
Fli1	14247	GSE22178	GSM552233	HaematopoieticProgenitors
Gata2	14461	GSE26031	GSM641911	HaematopoieticProgenitors
Gata2	14461	GSE22178	GSM552234	HaematopoieticProgenitors
Gfi1b	14582	GSE22178	GSM552235	HaematopoieticProgenitors
Ldb1	16825	GSE26031	GSM641909	HaematopoieticProgenitors
Lmo2	16909	GSE22178	GSM552237	HaematopoieticProgenitors
Lyl1	17095	GSE22178	GSM552238	HaematopoieticProgenitors
Meis1	17268	GSE22178	GSM552239	HaematopoieticProgenitors
Runx1	12394	GSE22178	GSM552241	HaematopoieticProgenitors
Runx1	12394	GSE31221	GSM773995-GSM773996	HaematopoieticProgenitors
Spi1	20375	GSE22178	GSM552240	HaematopoieticProgenitors
Tal1	21349	GSE26031	GSM641910	HaematopoieticProgenitors
Tal1	21349	GSE22178	GSM552242	HaematopoieticProgenitors
Tcf3	21423	GSE48086	GSM1167573	HaematopoieticProgenitors
Tcf7	21414	GSE31221	GSM773994	HaematopoieticProgenitors
Gfi1	14581	GSE50806	GSM1229967	ILC
Erg	13876	GSE46554	GSM1132365	Leukaemia
Erg	13876	GSE46554	GSM1132367	Leukaemia
Gfi1	14581	GSE31657	GSM786037	Leukaemia
Hoxa	111336	GSE33518	GSM842249-GSM842252	Leukaemia
Meis1	17268	GSE33518	GSM842248-GSM842251	Leukaemia
Mllt3	70122	GSE29130	GSM721212	Leukaemia
Mllt3	70122	GSE34261	GSM846042	Leukaemia
Notch1	18128	GSE29600	GSM732913	Leukaemia
Notch1	18128	GSE29600	GSM732916	Leukaemia
Notch1	18128	GSE34954	GSM859512	Leukaemia
Rbpj	19664	GSE29600	GSM732914	Leukaemia
Rbpj	19664	GSE29600	GSM732917	Leukaemia
Cebpa	12606	GSE21512	GSM537984	Macrophages
Cebpa	12606	GSE50565	GSM1223648	Macrophages

Cebpb	12608	GSE21512	GSM537985	Macrophages
JunB	16477	GSE38377	GSM1022318	Macrophages
JunB	16477	GSE38377	GSM1022319	Macrophages
Rela	19697	GSE16723	GSM611116-GSM611117	Macrophages
Rela	19697	GSE48759	GSM1183977	Macrophages
Rela	19697	GSE48759	GSM1183978	Macrophages
Spi1	20375	GSE21512	GSM537983	Macrophages
Spi1	20375	GSE38377	GSM940924-GSM940925	Macrophages
Spi1	20375	GSE38377	GSM1022260	Macrophages
Spi1	20375	GSE38377	GSM1022261	Macrophages
Spi1	20375	GSE38377	GSM1022262	Macrophages
Spi1	20375	GSE38377	GSM1022263	Macrophages
Spi1	20375	GSE38377	GSM1022280	Macrophages
Spi1	20375	GSE38377	GSM1022281	Macrophages
Spi1	20375	GSE38377	GSM1022282	Macrophages
Spi1	20375	GSE38377	GSM1022283	Macrophages
Spi1	20375	GSE38377	GSM1022310	Macrophages
Spi1	20375	GSE38377	GSM1022311	Macrophages
Spi1	20375	GSE38377	GSM1022312	Macrophages
Spi1	20375	GSE38377	GSM1022313	Macrophages
Spi1	20375	GSE38377	GSM940926	Macrophages
Spi1	20375	GSE38377	GSM940927	Macrophages
Spi1	20375	GSE38377	GSM940928	Macrophages
Spi1	20375	GSE38377	GSM940929	Macrophages
Spi1	20375	GSE38377	GSM940930	Macrophages
Spi1	20375	GSE38377	GSM940931	Macrophages
Spi1	20375	GSE38377	GSM940932	Macrophages
Spi1	20375	GSE38377	GSM940933	Macrophages
Spi1	20375	GSE38377	GSM940934	Macrophages
Spi1	20375	GSE48759	GSM1183968	Macrophages
Spi1	20375	GSE48759	GSM1183969	Macrophages
Spi1	20375	GSE48759	GSM1183972	Macrophages
Stat1	20846	GSE33913	GSM838681	Macrophages
Stat1	20846	GSE33913	GSM838683	Macrophages
Stat1	20846	GSE33913	GSM838685	Macrophages
Stat1	20846	GSE33913	GSM838687	Macrophages
Stat1	20846	GSE33913	GSM838689	Macrophages
Stat1	20846	GSE33913	GSM838691	Macrophages
Stat1	20846	GSE38377	GSM1022315	Macrophages
Stat1	20846	GSE38377	GSM1022316	Macrophages
Stat1	20846	GSE38377	GSM1022317	Macrophages
Stat6	20852	GSE38377	GSM1022301	Macrophages
Stat6	20852	GSE38377	GSM1022302	Macrophages
Stat6	20852	GSE38377	GSM1022303	Macrophages
Stat6	20852	GSE38377	GSM1022304	Macrophages
Stat6	20852	GSE38377	GSM1022305	Macrophages
Ctcf	13018	GSE48086	GSM1167574	Mast
Erg	13876	GSE48086	GSM1167576	Mast
Fli1	14247	GSE48086	GSM1167577	Mast
Fos	14281	GSE48086	GSM1167585	Mast
Gata2	14461	GSE42518	GSM1041378	Mast
Gfi1	14581	GSE42518	GSM1041379	Mast
Lmo2	16909	GSE48086	GSM1167579	Mast
Meis1	17268	GSE48086	GSM1167580	Mast
Mitf	17342	GSE48086	GSM1167584	Mast
Runx1	12394	GSE48086	GSM1167582	Mast
Spi1	20375	GSE48086	GSM1167581	Mast
Tal1	21349	GSE48086	GSM1167583	Mast
Tcf3	21423	GSE48086	GSM1167575	Mast
Ep300	328572	GSE45372	GSM1103356	Megakaryocyte
Runx1	12394	GSE45372	GSM1103355	Megakaryocyte
Cbfb	12400	GSE33653	GSM832312	MegakaryocyteProgenitors
Cbfb	12400	GSE33653	GSM832316	MegakaryocyteProgenitors
Ets1	23871	GSE31331	GSM777093	MegakaryocyteProgenitors
Gata1	14460	GSE31331	GSM777092	MegakaryocyteProgenitors
Gata1	14460	GSE36029	GSM923586	MegakaryocyteProgenitors
Gata1	14460	GSE35644	GSM872894	MegakaryocyteProgenitors
Gata2	14461	GSE31331	GSM777091	MegakaryocyteProgenitors
Rnf2	19821	GSE33653	GSM832313	MegakaryocyteProgenitors
Rnf2	19821	GSE33653	GSM832317	MegakaryocyteProgenitors
Rnf2	19821	GSE33653	GSM832320	MegakaryocyteProgenitors
Runx1	12394	GSE33653	GSM832311	MegakaryocyteProgenitors
Runx1	12394	GSE33653	GSM832315	MegakaryocyteProgenitors
Runx1	12394	GSE33653	GSM832319	MegakaryocyteProgenitors
Cbfa2t2	12396	ERA000161	ERX002140	MouseErythroLeukaemic

Cbfa2t2	12396	ERA000161	ERX002142	MouseErythroLeukaemic
Cbfa2t3	12398	ERA000161	ERX002126	MouseErythroLeukaemic
Cbfa2t3	12398	ERA000161	ERX002124	MouseErythroLeukaemic
Chd2	244059	GSE36030	GSM912929	MouseErythroLeukaemic
Ctcf	13018	GSE36029	GSM923573	MouseErythroLeukaemic
Ctcf	13018	GSE36030	GSM912896	MouseErythroLeukaemic
Ctcf	13018	GSE36027	GSM918744	MouseErythroLeukaemic
Ep300	328572	GSE36030	GSM912893	MouseErythroLeukaemic
Ep300	328572	GSE36030	GSM912921	MouseErythroLeukaemic
Gata1	14460	ERA000161	ERX002130	MouseErythroLeukaemic
Gata1	14460	ERA000161	ERX002128	MouseErythroLeukaemic
Gata1	14460	GSE36030	GSM912907	MouseErythroLeukaemic
Gata1	14460	GSE16594	GSM417015	MouseErythroLeukaemic
Gfi1b	14582	PMID22158964	PMID22158964	MouseErythroLeukaemic
JunD	16478	GSE36030	GSM912915	MouseErythroLeukaemic
Ldb1	16825	ERA000161	ERX002134	MouseErythroLeukaemic
Ldb1	16825	ERA000161	ERX002132	MouseErythroLeukaemic
Mafk	17135	GSE36030	GSM912899	MouseErythroLeukaemic
Max	17187	GSE36030	GSM912919	MouseErythroLeukaemic
Mxi1	17859	GSE36030	GSM912928	MouseErythroLeukaemic
Myb	17863	GSE36030	GSM912903	MouseErythroLeukaemic
Myc	17869	GSE36030	GSM912934	MouseErythroLeukaemic
Rad21	19357	GSE36030	GSM912933	MouseErythroLeukaemic
Rad21	19357	GSE36030	GSM912935	MouseErythroLeukaemic
Rdbp	27632	GSE36030	GSM912932	MouseErythroLeukaemic
Smc3	13006	GSE36030	GSM912923	MouseErythroLeukaemic
Tal1	21349	ERA000161	ERX002138	MouseErythroLeukaemic
Tal1	21349	ERA000161	ERX002136	MouseErythroLeukaemic
Tal1	21349	GSE36029	GSM923578	MouseErythroLeukaemic
Irf8	15900	GSE38824	GSM950325	MyeloidProgenitors
Myb	17863	GSE22095	GSM549337-GSM549339-GSM549341	MyeloidProgenitors
Spi1	20375	GSE38824	GSM1031977	MyeloidProgenitors
Batf	53314	GSE40918	GSM1004790-GSM1004792	T-Cells
Batf	53314	GSE40918	GSM1004789-GSM1004791	T-Cells
Batf	53314	GSE40918	GSM1004784-GSM1004785	T-Cells
Batf	53314	GSE40918	GSM1004795-GSM1004797	T-Cells
Batf	53314	GSE40918	GSM1004794-GSM1004796	T-Cells
Batf	53314	GSE40918	GSM1004786-GSM1004787	T-Cells
Batf	53314	GSE40918	GSM1004793	T-Cells
Batf	53314	GSE39756	GSM978758	T-Cells
Batf	53314	GSE39756	GSM978767	T-Cells
Batf	53314	GSE54191	GSM1309509-GSM1309510	T-Cells
Ctcf	13018	GSE40918	GSM1004802-GSM1004803	T-Cells
Ctcf	13018	GSE40918	GSM1004804-GSM1004805	T-Cells
Egr2	13654	GSE49366	GSM1198167	T-Cells
Elf1	13709	GSE40684	GSM999184	T-Cells
Elf1	13709	GSE40684	GSM999185	T-Cells
Ep300	328572	GSE40463	GSM994508-GSM994529	T-Cells
Ep300	328572	GSE40463	GSM994516-GSM994533	T-Cells
Ep300	328572	GSE40918	GSM1004841-GSM1004842	T-Cells
Ep300	328572	GSE40918	GSM1004849-GSM1004850	T-Cells
Ep300	328572	GSE40918	GSM1004843	T-Cells
Ep300	328572	GSE40918	GSM1004844	T-Cells
Ep300	328572	GSE40918	GSM1004845	T-Cells
Ep300	328572	GSE40918	GSM1004846	T-Cells
Ep300	328572	GSE40918	GSM1004847	T-Cells
Ep300	328572	GSE40918	GSM1004848	T-Cells
Ep300	328572	GSE40918	GSM1004851	T-Cells
Ep300	328572	GSE40918	GSM1004852	T-Cells
Ep300	328572	GSE40463	GSM994524	T-Cells
Ep300	328572	GSE40463	GSM994520	T-Cells
Ets1	23871	GSE40684	GSM999186	T-Cells
Ets1	23871	GSE40684	GSM999187	T-Cells
Etv6	14011	GSE40918	GSM1004806-GSM1004807	T-Cells
Fli1	14247	GSE20898	GSM654874	T-Cells
Fosl2	14284	GSE40918	GSM1004809-GSM1004810	T-Cells
Fosl2	14284	GSE40918	GSM1004808	T-Cells
Foxo1	56458	GSE40656	GSM998924	T-Cells
Foxo1	56458	GSE46525	GSM1131775	T-Cells
Foxp3	20371	GSE40684	GSM999179-GSM999180-GSM999182	T-Cells
Gata3	14462	GSE20898	GSM523221	T-Cells
Gata3	14462	GSE20898	GSM523222	T-Cells
Gata3	14462	GSE20898	GSM523223	T-Cells
Gata3	14462	GSE20898	GSM523224	T-Cells
Gata3	14462	GSE20898	GSM523225	T-Cells

Gata3	14462	GSE20898	GSM523226	T-Cells
Gata3	14462	GSE20898	GSM523228	T-Cells
Gata3	14462	GSE20898	GSM523229	T-Cells
Gata3	14462	GSE20898	GSM523230	T-Cells
Gata3	14462	GSE20898	GSM523231	T-Cells
Gata3	14462	GSE31235	GSM774295	T-Cells
Gata3	14462	GSE31235	GSM774296	T-Cells
Gata3	14462	GSE31235	GSM774297	T-Cells
Hif1a	15251	GSE40918	GSM1004819-GSM1004820	T-Cells
Irf4	16364	GSE40918	GSM1004823-GSM1004824	T-Cells
Irf4	16364	GSE40918	GSM1004834-GSM1004836	T-Cells
Irf4	16364	GSE40918	GSM1004833-GSM1004835	T-Cells
Irf4	16364	GSE40918	GSM1004821	T-Cells
Irf4	16364	GSE40918	GSM1004822	T-Cells
Irf4	16364	GSE40918	GSM1004825	T-Cells
Irf4	16364	GSE40918	GSM1004826	T-Cells
Irf4	16364	GSE40918	GSM1004827	T-Cells
Irf4	16364	GSE40918	GSM1004828	T-Cells
Irf4	16364	GSE40918	GSM1004829	T-Cells
Irf4	16364	GSE40918	GSM1004830	T-Cells
Irf4	16364	GSE40918	GSM1004831	T-Cells
Irf4	16364	GSE40918	GSM1004837	T-Cells
Irf4	16364	GSE40918	GSM1004838	T-Cells
Irf4	16364	GSE39756	GSM978750	T-Cells
Irf4	16364	GSE39756	GSM978751	T-Cells
Irf4	16364	GSE39756	GSM978768	T-Cells
Irf4	16364	GSE54191	GSM1309511-GSM1309512	T-Cells
Jun	16476	GSE39756	GSM978770	T-Cells
Jun	16476	GSE54191	GSM1309513	T-Cells
JunB	16477	GSE39756	GSM978769	T-Cells
JunB	16477	GSE54191	GSM1309514	T-Cells
JunD	16478	GSE39756	GSM978771	T-Cells
JunD	16478	GSE54191	GSM1309515	T-Cells
Kdm6b	216850	GSE40918	GSM1004839	T-Cells
Kdm6b	216850	GSE40918	GSM1004840	T-Cells
Maf	17132	GSE40918	GSM1004799-GSM1004800	T-Cells
pStat3	20848	GSE21669	GSM540722	T-Cells
Rorc	19885	GSE40918	GSM1004855-GSM1004856	T-Cells
Spi1	20375	GSE31235	GSM774291	T-Cells
Spi1	20375	GSE31235	GSM774292	T-Cells
Spi1	20375	GSE31235	GSM774293	T-Cells
Stat1	20846	GSE40463	GSM994528	T-Cells
Stat3	20848	GSE26552	GSM652877	T-Cells
Stat3	20848	GSE40918	GSM1004864-GSM1004866	T-Cells
Stat3	20848	GSE40918	GSM1004863-GSM1004865	T-Cells
Stat3	20848	GSE40918	GSM1004860-GSM1004861	T-Cells
Stat3	20848	GSE40918	GSM1004858	T-Cells
Stat3	20848	GSE40918	GSM1004859	T-Cells
Stat3	20848	GSE39756	GSM978757	T-Cells
Stat3	20848	GSE39756	GSM978772	T-Cells
Stat4	20849	GSE22104	GSM550303	T-Cells
Stat5a	20850	GSE36882	GSM904760	T-Cells
Stat5a	20850	GSE36882	GSM904766	T-Cells
Stat5a/b	20850	GSE26552	GSM652878	T-Cells
Stat5b	20851	GSE36882	GSM904761	T-Cells
Stat5b	20851	GSE36882	GSM904767	T-Cells
Stat5b	20851	GSE41317	GSM1014575	T-Cells
Stat5b	20851	GSE41317	GSM1234366	T-Cells
Stat5b	20851	GSE41317	GSM1234369	T-Cells
Stat5b	20851	GSE41317	GSM1234370	T-Cells
Stat6	20852	GSE22104	GSM550311	T-Cells
Stat6	20852	GSE41317	GSM1014576	T-Cells
Stat6	20852	GSE41317	GSM1234371	T-Cells
Stat6	20852	GSE41317	GSM1234372	T-Cells
Tbx21	57765	GSE33802	GSM836124	T-Cells
Cbfb	12400	GSE33653	GSM832322	Thymocytes
Ctcf	13018	GSE41743	GSM1023416	Thymocytes
Ctcf	13018	GSE41743	GSM1023418	Thymocytes
Ctcf	13018	GSE36027	GSM918734	Thymocytes
Hnf1a	21405	GSE46662	GSM1133644	Thymocytes
Rag2	19374	GSE21207	GSM530318	Thymocytes
Rnf2	19821	GSE33653	GSM832323	Thymocytes
Runx1	12394	GSE33653	GSM832321	Thymocytes
